# Risk Score Prediction Model of Prognosis in GC Patients by Age and Gender Combined With m6A Modification Genes FTO and RBM15

**DOI:** 10.3389/fcell.2022.710708

**Published:** 2022-03-31

**Authors:** Limin Yue, Rongguang Zhang, Shuaiyin Chen, Guangcai Duan

**Affiliations:** ^1^ Department of Epidemiology, College of Public Health, Zhengzhou University, Zhengzhou, China; ^2^ Department of Epidemiology, College of Public Health, Hainan Medical University, Haikou, China

**Keywords:** gastric cancer, N6-methyladenosine, prognostic factor, FTO, RBM15

## Abstract

**Background:** Gastric cancer (GC) has a high mortality rate. N6-methyladenosine (m6A) is involved in the development of GC. Age and gender are associated with GC incidence and survival. This study aimed to explore the risk score prediction model of prognosis in GC patients by age and gender combined with m6A modification genes.

**Methods:** Data on m6A modification gene expression and clinical information downloaded from the Cancer Genome Atlas (TCGA) database were used to construct the risk score prediction model. Cox and least absolute shrinkage and selection operator (LASSO) regression were performed to identify clinical characteristics and m6A modification genes associated with prognosis. A risk score prediction model was established based on multivariate Cox regression analysis. The Gene Expression Omnibus (GEO) database was used to validate this model.

**Results:** Most of the m6A modification genes were upregulated in GC tumor tissues compared with that in normal tissues and were correlated with clinical characteristics including grade, stage status, and T status. The risk score prediction model was established based on age, gender, FTO, and RBM15. GC patients were divided into high- or low-risk groups based on the median risk score. Patients with a high risk score had poor prognosis. Multivariate Cox regression indicated that risk score was an independent prognostic factor for GC patients. The data from GSE84437 verified the predictive value of this model.

**Conclusion:** The risk score prediction model based on age and gender combined with m6A modification genes FTO and RBM15 was an independent prognostic factor for GC patients.

## Introduction

Gastric cancer (GC) is the fourth most common cancer in the world and the second leading cause of death (Siegel et al., 2020). Age is a risk factor for GC. The incidence of GC increases with age and is two times higher in male than in female population (Bray et al., 2018), and more than 75% of affected people are older than 50 years (Kranjcevic et al., 2015) . The occurrence and development of GC is a complex multistep process involving a large number of genetic and epigenetic changes (Zhang et al., 2019b).

N6-methyladenosine (m6A) modification refers to the methylation of the six nitrogen position of mRNA adenosine, which mainly occurs in the common sequence of RRACH (R = G or A, H = A, C, or U) (Csepany et al., 1990; Narayan et al., 1994; Niu et al., 2013). m6A is an abundant nucleotide modification in eukaryotic mRNA (Desrosiers et al., 1974). m6A modification dynamically encodes RNA by “writers” and “erasers” and then decodes the RNA by “readers,” thus causing subsequent biological function changes. Writers mediate the process of RNA methylation modification, including METTL3, METTL14, METTL16, RBM15, WTAP, KIAA1429, and ZC3H13 (Scholler et al., 2018), while erasers mediate the process of RNA demethylation modification, including FTO and ALKBH5 (Huang et al., 2015). Readers can read methylated RNA and participate in the translation and degradation of downstream RNA, including YTHDC1, YTHDC2, YTHDF1, YTHDF2, YTHDF3, IGF2BP1, IGF2BP2, IGF2BP3, HNRNPA2B1, and HNRNPC (Wojtas et al., 2017).

It is reported that m6A methylation is a dynamic reversible process (Fu et al., 2014), which has been confirmed with various diseases, such as neuronal diseases (Angelova et al., 2018), diabetes (Shen et al., 2015), obesity (Ben-Haim et al., 2015), and tumor (J. Chen and Du, 2019). However, the combined effects of age, gender, and m6A modification genes on GC remain unclear. This study aimed to explore the relationship between clinical characteristics and m6A modification genes and prognosis of GC, and to build a prognosis model of GC patients combining clinical characteristics and m6A modification genes.

## Methods

### Data Acquisition

The RNA-seq transcriptome profiling and clinical information of STAD patients were downloaded from the TCGA (https://portal.gdc.cancer.gov/) and GEO (https://www.ncbi.nlm.nih.gov/geo/) online databases. The data from the TCGA database were used to construct the risk score prediction model for prognosis of GC patients, and the data from GSE84437 were used to validate the risk score prediction model.

Perl and R were utilized to process the data downloaded from the TCGA database and GEO online database GSE84437.

### Bioinformatic Analysis

The R package “limma,” “pheatmap,” and “vioplot” visualized the expression of m6A modification genes in tumor and adjacent normal tissues. The different genes differing in expression between tumor and normal tissues were defined with *p* < 0.05. R package “corrplot” was used to analyze and visualize the relationship between every two genes. Multivariate Cox regression was used to identify factors related to GC prognosis. Least absolute shrinkage and selection operator (LASSO) Cox regression was used to test the collinearity of variables screened by multivariate Cox regression and to reduce the dimensionality of these variables (B. Wang et al., 2022; C. Xu et al., 2019). After dimensionality reduction through LASSO Cox regression, the variables were included in the multivariate Cox regression. Then, the coefficients obtained by multivariate Cox regression analysis were used to establish the risk score prediction model for predicting the prognosis of GC patients from the TCGA database. The formula was established as follows: risk score = sum (each gene’s expression × corresponding coefficient). The patients were stratified into high- or low-risk groups based on the median value of the risk score.

The Kaplan–Meier plotter website (http://www.kmplot.com) was used to verify the association between the expression of genes obtained from the multivariate Cox regression analysis and the survival of GC patients. The threshold was adjusted to *p* < 0.05.

To test the robustness of the risk score prediction model constructed from the TCGA cohort, the risk score for each GC patient from GEO online database GSE84437 was calculated using the same formula as that used for the TCGA cohort. The patients from GSE84437 were also categorized into high- or low-risk groups by the median risk score.

### Statistical Analysis

Chi-square test and t-test were used to assess the association of risk score with clinical characteristics. Spearman’s rank correlation, one-way ANOVA, and t-test were used to explore the relationship between m6A modification genes and clinical characteristics.

Using R version 3.6 for all statistical analyses, *p* < 0.05 was statistically significant.

## Results

### RNA m6A Modification Genes in Gastric Cancer

**FIGURE 1:**
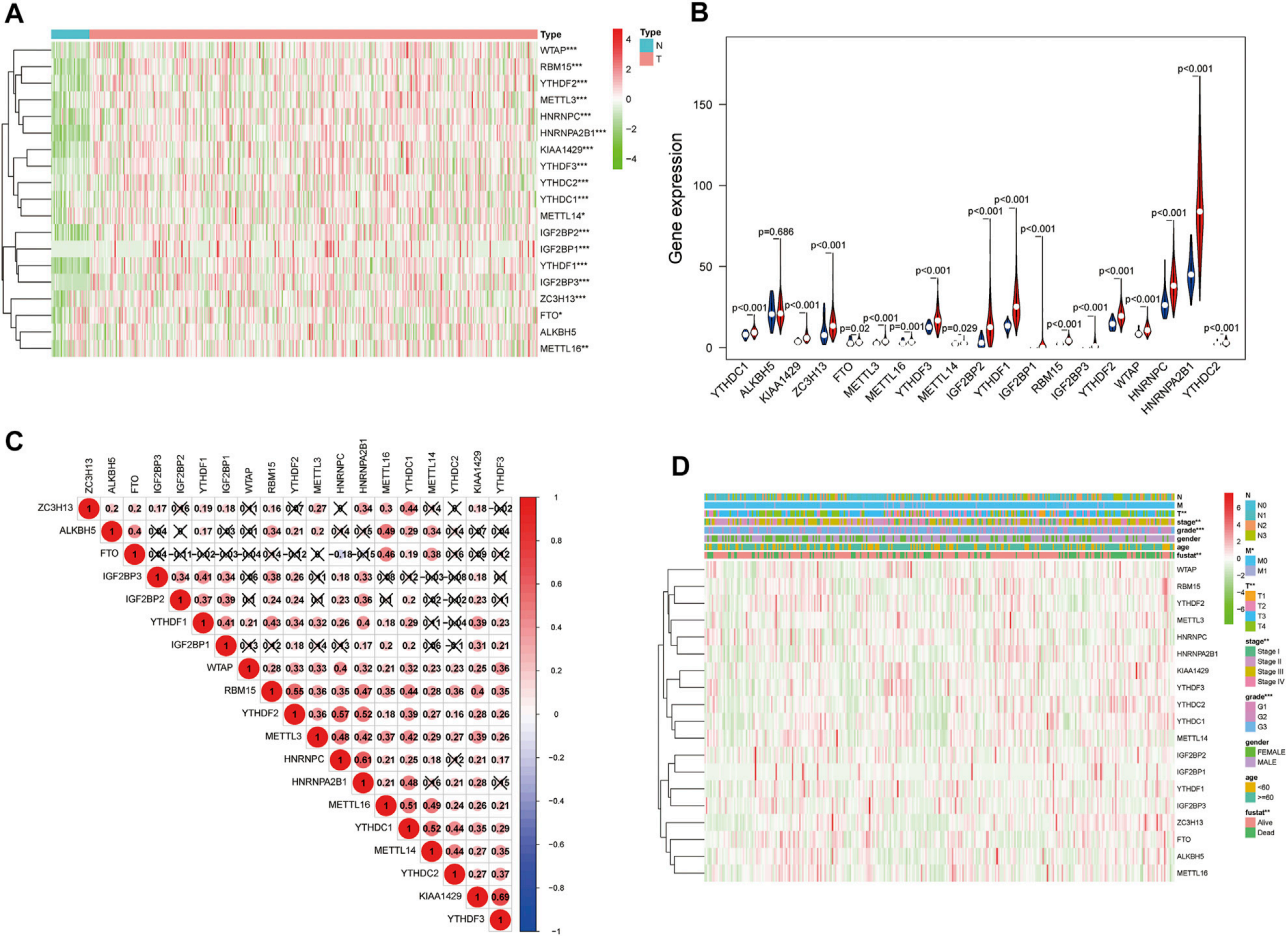
m6A modification genes in GC in the TCGA database. **(A)** Heatmap shows the expression of the 19 m6A modification genes in 375 GC and 32 adjacent normal tissues. The higher or lower the expression, the darker the color; red represents upregulated, and green, downregulated m6A modification genes. **(B)** Vioplot visualizing the differentially expressed m6A modification genes in different tissue samples in GC; blue represents adjacent normal tissues, and red, GC tissues). **(C)** Spearman correlation analysis of the 19 m6A modification genes in GC. **(D)** Expression of m6A modification genes in GC with different clinicopathological features. *p < 0.05, **p < 0.01, and ***p < 0.001. GC, gastric cancer.

The expression of the 19 m6A modification genes was analyzed in 375 GC tumor tissues and 32 adjacent normal tissues in the TCGA database. The result showed that compared with the normal tissues, the tumor tissues in GC patients had a higher expression of m6A modification genes (except ALKBH5) (Figures 1A,B). As shown in Figure 1C, the 19 m6A modification genes were weakly to moderately correlated. The HNRNPC and FTO genes were negatively correlated, while most of the other genes were positively correlated. Correlation analysis of these 19 m6A modification genes with clinical characteristics indicated that these genes were related to survival status, grade, stage status, and T status (Figure 1D; Supplementary Figures S1A–D; Supplementary Figures S2A–D).

### Influence of m6A Modification Genes and Clinical Characteristics on Prognosis of Gastric Cancer Patients

**FIGURE 2:**
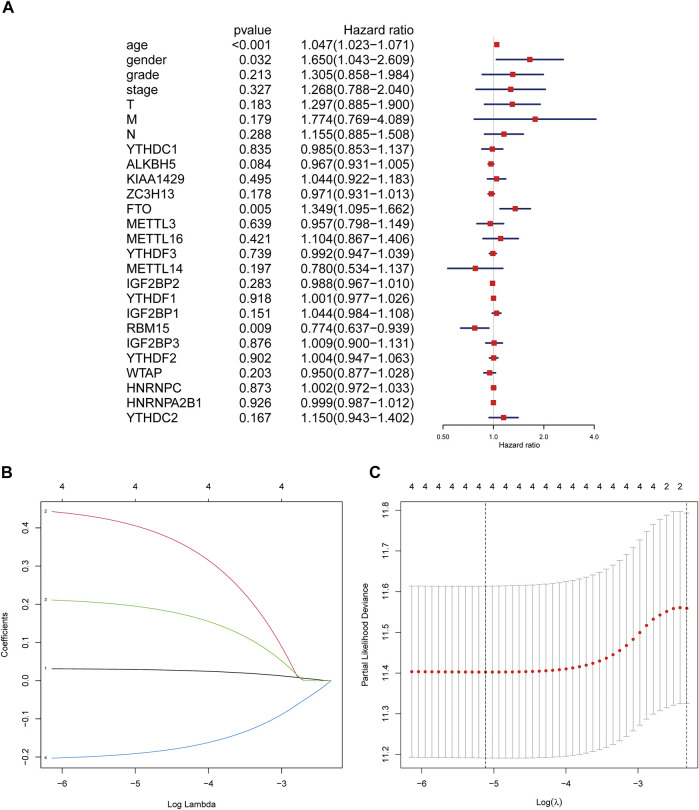
Regression analysis of the relationship between clinical characteristics and m6A modification genes and prognosis of GC patients in the TCGA database. **(A)** Multivariate Cox regression analysis of the prognosis in GC patients. **(B)** LASSO coefficient profiles of the expression of 2 m6A modification genes including FTO and RBM15 and two clinical characteristics including age and gender. **(C)** Selection of the penalty parameter (*λ*) in the LASSO Cox regression via 10-fold cross-validation. The dotted vertical lines are plotted at the optimal values following the minimum criteria (left) and “one standard error” criteria (right). GC, gastric cancer.

**FIGURE 3:**
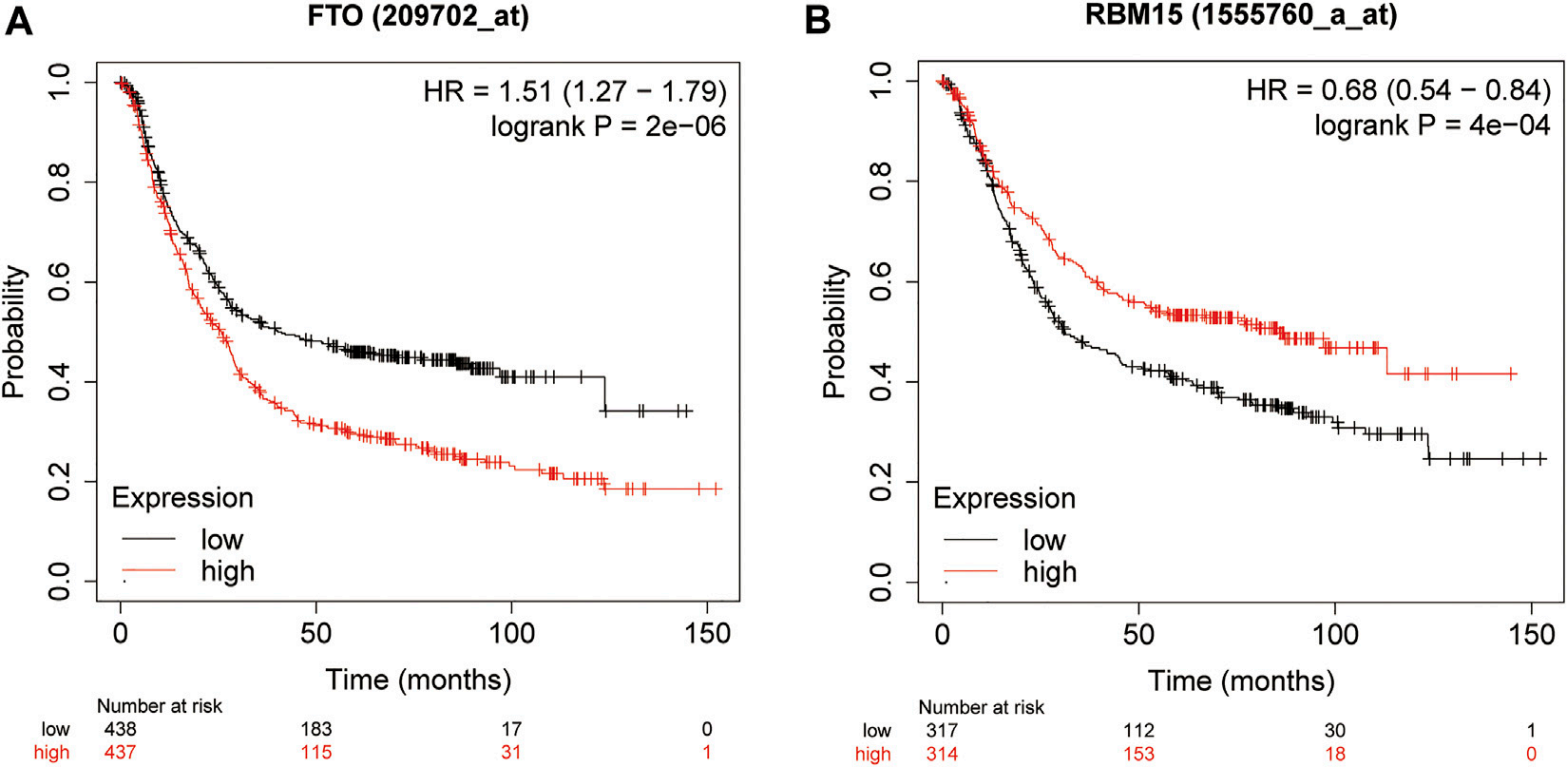
Kaplan–Meier overall survival curves for GC patients using the online Kaplan–Meier plotter survival analysis (http://www.kmplot.com) based on the median expression of FTO **(A)** and RBM15 **(B)**. GC, gastric cancer.

In order to explore the prognostic factors of GC patients, patients lacking clinical or survival information were excluded from the prognostic analysis. A total of 317 GC patients were included in the following analysis. The clinical characteristics of 317 GC patients from the TCGA database are shown in Supplementary Table S1. Multivariate Cox regression was used to analyze the influence of m6A modification genes and clinical characteristics on the prognosis of GC patients. Multivariate Cox regression analysis showed that age (HR = 1.05, 95% CI: 1.02–1.07), gender (HR = 1.65, 95% CI: 1.04–2.61), FTO (HR = 1.35, 95% CI: 1.10–1.66), and RBM15 (HR = 0.77, 95% CI: 0.64–0.94) were associated with the prognosis of GC patients (Figure 2A). Through dimension reduction analysis of the four variables, LASSO Cox regression still obtained four variables, including two clinical characteristics age and gender and two m6A modification genes FTO and RBM15 (Figures 2B,C). The online Kaplan–Meier plotter survival analysis tool (http://www.kmplot.com) was used to verify the genes FTO and RBM15 related to the prognosis of GC. The results also showed that FTO (HR = 1.51, 95% CI: 1.27–1.79) was a risk factor and RBM15 (HR = 0.68, 95% CI: 0.54–0.84) was a protective factor for GC prognosis (Figures 3A,B).

### Risk Score Prediction Model of Prognosis in GC Patients by Age and Gender Combined With m6A Modification Genes FTO and RBM15

LASSO Cox regression was used to test the collinearity of variables screened by multivariate Cox regression and to reduce the dimensionality of these variables (B. Wang et al., 2022; C. Xu et al., 2019). After dimensionality reduction through LASSO Cox regression, the four variables age, gender, FTO, and RBM15 were included in the multivariate Cox regression. Then, the coefficients obtained using the multivariate Cox regression analysis were used to establish the risk score prediction model for predicting the prognosis of GC patients from the TCGA database. Regression coefficients and *p* values are shown in Table 1.

**TABLE 1:** Regression coefficients and *p* values obtained by multivariate Cox regression in GC patients (from TCGA survival data).

Variable	Regression coefficients	*p* Values
Age	0.046	0.001
Gender	0.501	0.032
FTO	0.300	0.003
RBM15	−0.257	0.001

GC, gastric cancer.

**FIGURE 4:**
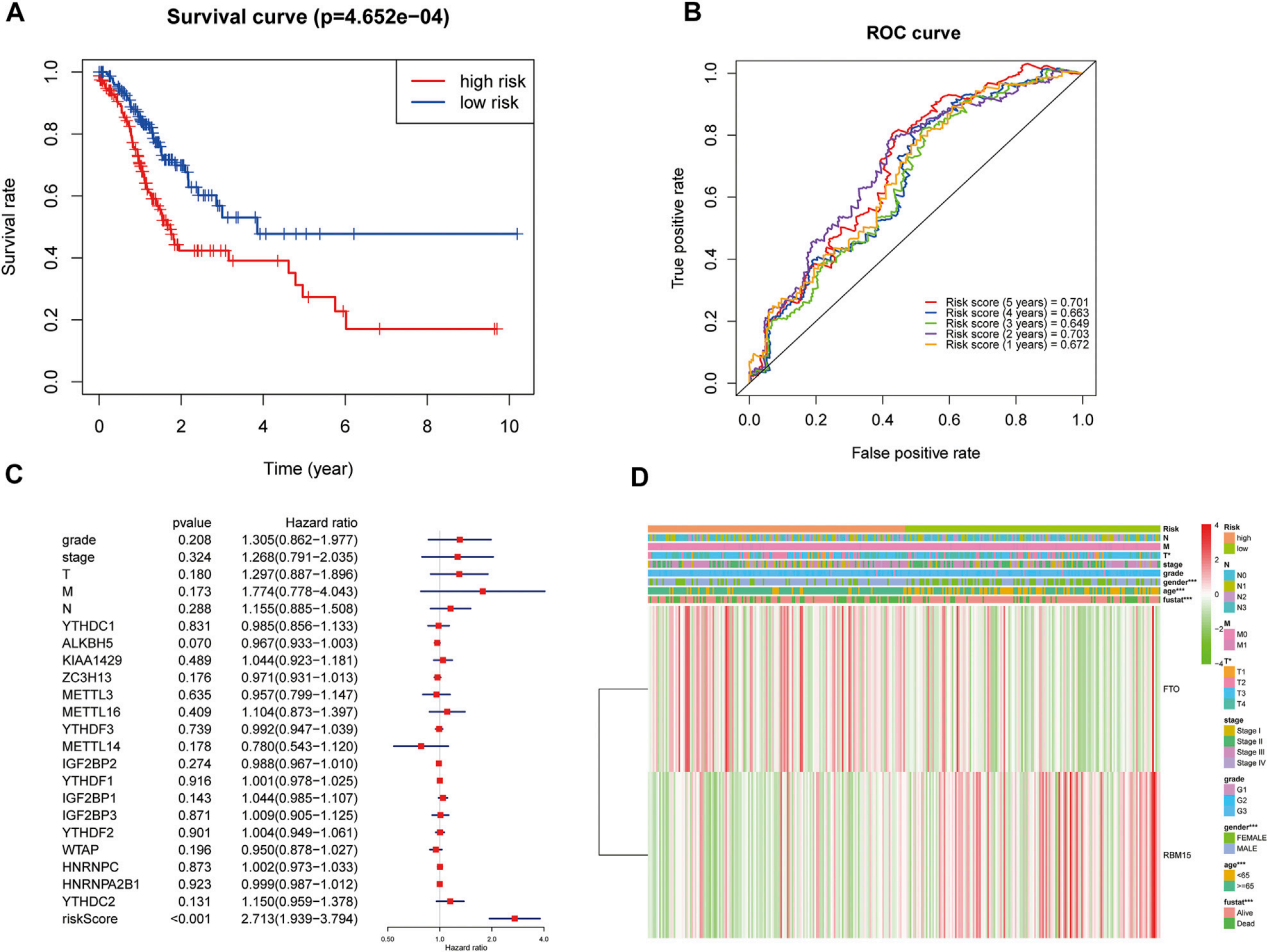
Relationship between the risk score, clinical characteristics, and cluster subgroups. **(A)** Kaplan–Meier overall survival curves for patients in the TCGA database assigned to high- and low-risk groups based on the median risk score. **(B)** ROC curves showing the predictive efficiency of the risk score signature. **(C)** Multivariate Cox regression analyses of the association between clinical characteristics (including the risk score) and overall survival of GC patients in the TCGA database. **(D)** Heatmap showing the expression of FTO and RBM15 in low and high risk score patients with GC. Distribution of the clinical characteristics was compared between the low- and high-risk groups. *p < 0.05 and ***p < 0.001. GC, gastric cancer.

So the combination prognostic model of age and gender combined with m6A modification genes FTO and RBM15 for GC patients was obtained: “risk score = 0.046 × age + 0.501 × gender + 0.300 × FTO − 0.257 × RBM15.” According to this formula, everyone had a risk score. The patients were divided into high- or low-risk groups based on the median risk score. The Kaplan–Meier plotter showed that GC patients with a high risk score had a bad prognosis (Figure 4A, *p* < 0.001).

The ROC (receiver operating characteristic) was used to evaluate the risk score prediction model in predicting survival. As shown in Figure 4B, the areas under the ROC curve (AUC) were 0.701, 0.663, 0.649, 0.703, and 0.672 in the 5th, 4th, 3rd, 2nd, and 1st years, respectively, which indicated that the risk score can predict survival in GC patients. Multivariate Cox regression showed that risk score was an independent risk factor for poor prognosis in GC patients (HR = 2.71, 95% CI:1.94–3.79, Figure 4C). Then the association of the risk score with clinical characteristics was assessed. The results showed that T status (*p* < 0.05), gender (*p* < 0.001), age (*p* < 0.001), and prognosis (*p* < 0.001) were related to the risk score (Figure 4D). Moreover, compared with GC patients in the low risk score group, patients in the high risk score group had higher expression of FTO and lower expression of RBM15 (Figure 4D).

### Validation of the Risk Score Prediction Model of Age and Gender Combined With m6A Modification Genes FTO and RBM15

**FIGURE 5:**
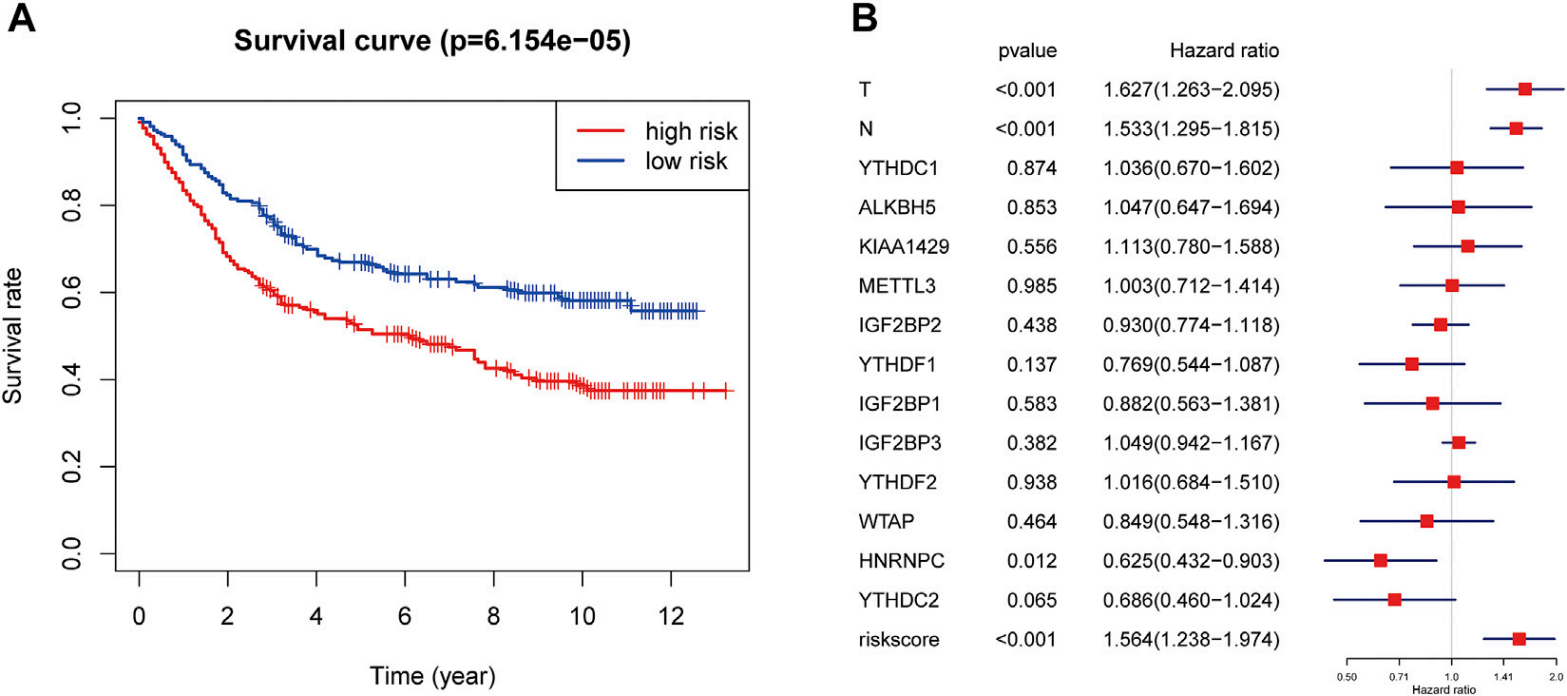
Validation of the risk score prediction model using GEO database GSE84437. **(A)** Kaplan–Meier overall survival curves for patients in the GSE84437 database assigned to high- and low-risk groups based on the median risk score. **(B)** Multivariate Cox regression analyses of the association between clinical characteristics (including the risk score) and overall survival of GC patients in the GSE84437 database. GC, gastric cancer.

GEO database GSE84437 was used to validate the risk score prediction model. The clinical characteristics of 433 GC patients from GSE84437 are shown in Supplementary Table S2. The risk score for each GC patient from GSE84437 was calculated using the formula that was used for the TCGA cohort. All patients were divided into high- or low-risk groups according to the median risk score. The Kaplan–Meier plotter showed that GC patients with a high risk score had a worse prognosis (Figure 5A, *p* < 0.001). Multivariate Cox regression also suggested that risk score was an independent risk factor for worse prognosis in GC patients (HR = 1.56, 95% CI:1.24–1.97, Figure 5B).

## Discussion

Gastric cancer is a serious threat to human health. Although the global incidence of GC has decreased significantly in recent decades, the 5-year survival rate of GC is still below 30%, and many critical issues remain to be addressed (Hironaka, 2019). Increasing numbers of studies have elucidated the genetic and epigenetic mechanisms in the progression of GC, but the pathogenesis of GC is still not fully understood (Raimondi et al., 2018). RNA modification is a kind of gene modification with genetic and dynamic characteristics that remain throughout life, which is a research area of great concern (Roundtree et al., 2017). m6A is a type of RNA modification that is closely related to the occurrence and development of GC (Yue et al., 2019; Mei et al., 2020).

As a member of the epigenetic family, m6A can regulate tumor cells mainly by regulating the mRNA expression of related oncogenes or tumor suppressor genes. The m6A methylation site of nuclear RNA appears under the action of “writers,” and this site can also be erased by the “erasers.” Subsequently, during further processing of the nuclear RNA, the readers in the nucleus will bind to the m6A methylation site. When the mature RNA is released from the nucleus, some extracellular readers will bind to the m6A methylation site (Su et al., 2019). The m6A methylation level is closely associated with the expression level of “writers” and “erasers,” and different “readers” bind to the m6A methylation site to produce a series of biological functions (X. Y. Chen et al., 2019; Sun et al., 2019).

m6A “writers” include METTL3, METTL14, METTL16, RBM15, WTAP, KIAA1429, and ZC3H13 (Scholler et al., 2018). FTO and ALKBH5 are m6A “erasers” (Huang et al., 2015). M6A “readers” include YTHDC1, YTHDC2, YTHDF1, YTHDF2, YTHDF3, IGF2BP1, IGF2BP2, IGF2BP3, HNRNPA2B1, and HNRNPC (Wojtas et al., 2017). These m6A modification genes were used as 19 candidate genes for differential expression analysis. The present study not only found that the m6A modification genes were disorderly expressed in GC but also found that the m6A modification genes were related to the clinicopathological characteristics. Eighteen m6A modification genes were found to be upregulated in GC, including the “writers”: METTL3, METTL14, METTL16, RBM15, WTAP, KIAA1429, and ZC3H13; “eraser”: FTO; and the “readers”: YTHDC1, YTHDC2, YTHDF1, YTHDF2, YTHDF3, IGF2BP1, IGF2BP2, IGF2BP3, HNRNPA2B1, and HNRNPC. The results suggested that the dysregulated m6A modification genes might promote the occurrence and development of GC. For example, high expression of METTL3 could promote the progression of GC (Jiang et al., 2020; Q.; Wang et al., 2020). Yang et al. reported that FTO expression was upregulated in GC tissues and promoted GC cell proliferation, migration, and invasion by activating the MYC signaling pathway (Z. Yang et al., 2021). Overexpression of FTO reduced m6A modification and contributed to the malignant phenotype of GC by promoting the activation of the Wnt/PI3K-Akt pathway (Zhang et al., 2019a). In addition, FTO was involved in the occurrence and prognosis of GC (D. Xu et al., 2017). Jing et al. also found that RBM15 was upregulated in GC (Jing et al., 2021). The high expression of RBM15 could stimulate Notch signaling by RBPJk, and the enhancement of Notch signaling was associated with hematological malignancies. Reducing RBM15 expression level by RNA interference could inhibit the growth and proliferation of chronic myelogenous leukemia cells, block the cell cycle, and induce apoptosis (Y. Yang et al., 2012). RBM15 also contributed to chromosomal translocation in acute megakaryocytic leukemia (Mercher et al., 2001).

Studies reported that GC incidence rate was associated with age (Crew and Neugut, 2006; Liang et al., 2013). Hu et al. and Chen et al. suggested that age was a risk factor for poor survival in GC (J. Chen et al., 2016; Hu et al., 2019). Lou et al. reported that male gender had higher GC incidence than female gender (Lou et al., 2020). Li et al. suggested that in GC patients, the survival prognosis for female patients was better than that for male patients (Li et al., 2019). In addition to clinical characteristics, m6A modification genes were also associated with prognosis in GC patients. For example, high FTO expression was significantly associated with poor prognosis in GC patients (D. Xu et al., 2017). In this study, not only age and gender but also FTO and RBM15 were found to be independent prognostic factors in GC patients. Some studies also reported that high expression of FTO was significantly associated with worse prognosis in GC, and high expression of RBM15 was significantly related to better prognosis in GC (Su et al., 2019; Zhang et al., 2020).

The expression of RBM15 in GC tissues was higher than that in normal tissues, but the prognosis of patients with high RBM15 expression was significantly better than that of those with low RBM15 expression. The possible explanation is that patients with high RBM15 expression were more sensitive to chemotherapy. The results of the TCGA database in this study showed that in GC patients receiving chemotherapy, patients with high RBM15 expression had better prognosis than those with low RBM15 expression (HR = 0.72, 95% CI: 0.57–0.91). Considering that chemotherapy had no effect on the prognosis of GC patients in the TCGA database (*p* = 0.18), and a large number of patients did not report chemotherapy information, chemotherapy variable was not included in the multivariate Cox regression and LASSO regression in this study.

Currently, the epigenetics of RNA is receiving increasing attention and is becoming an attractive field of study. Many studies have established prognostic models based on the expression of m6A modification genes in a variety of tumors, including GC. However, a prognostic model combining m6A modification genes with clinical characteristics has not been found. Therefore, this study used the TCGA database to establish a risk score prediction model of prognosis in GC patients based on age, gender, FTO, and RBM15. The risk score prediction model had important clinical significance in predicting the prognosis of GC patients. Patients with GC in the high-risk group had worse prognosis than those in the low-risk group. Such a prognosis score can be used in clinical practice to facilitate treatment selection. In addition, GEO database GSE84437 was used to verify the validity of the prediction model.

However, this study has some limitations. First, the construction and validation of the risk score prediction model in this study were carried out using an open database. The validity of this model needs to be verified in a larger sample size cohort study. Second, the specific mechanism of involvement of m6A modification genes in the occurrence and development of GC still needs to be further studied *in vivo* and *in vitro*.

## Conclusion

The expression of m6A modification genes was found to be disordered in GC and play an important role in the prognosis of GC patients. The risk score prediction model based on age and gender combined with m6A modification genes FTO and RBM15 was an independent prognostic factor of GC. m6A modification gene expression may become a potential marker for tumor molecular diagnosis and will also provide new targets for the development of clinical molecular targeted therapeutic drugs.

## Data Availability

Publicly available datasets were analyzed in this study. These data can be found at: https://portal.gdc.cancer.gov/ and https://www.ncbi.nlm.nih.gov/geo/query/acc.cgi?acc=GSE84437.
