# Supplementary material for: Risk Score Prediction Model of Prognosis in GC Patients by Age and Gender Combined With m6A Modification Genes FTO and RBM15
Source: Front Cell Dev Biol. 2022 Mar 31;10:710708. doi: 10.3389/fcell.2022.710708 (PMC9008303; doi:10.3389/fcell.2022.710708)
Supplement: Supplementary file 1 [file DataSheet1.pdf]

Supplementary materials

Title:

Risk score prediction model of prognosis in GC patients by age and gender combined with m6A modification genes FTO and RBM15

Limin Yue[1], Rongguang Zhang[1, 2*], Shuaiyin Chen[1*], Guangcai Duan[1]

[1]Department of Epidemiology, College of Public Health, Zhengzhou University

[2]Department of Epidemiology, College of Public Health, Hainan Medical University

Rongguang Zhang and Shuaiyin Chen are the co-corresponding authors.

***Corresponding Author:**

Rongguang Zhang. Email: zrg@zzu.edu.cn, Department of Epidemiology, College of Public Health, Zhengzhou University, No.100 Kexue Avenue, Zhengzhou 450001, China; Department of Epidemiology, College of Public Health, Hainan Medical University, No.3 Xueyuan Road, Longhua District, Haikou 570216, China. ORCID: 0000-0001-9961-2434.

Shuaiyin Chen. Email: sychen@zzu.edu.cn, Department of Epidemiology, College of Public Health, Zhengzhou University, No.100 Kexue Avenue, Zhengzhou 450001, China. ORCID: 0000-0001-6129-0310.

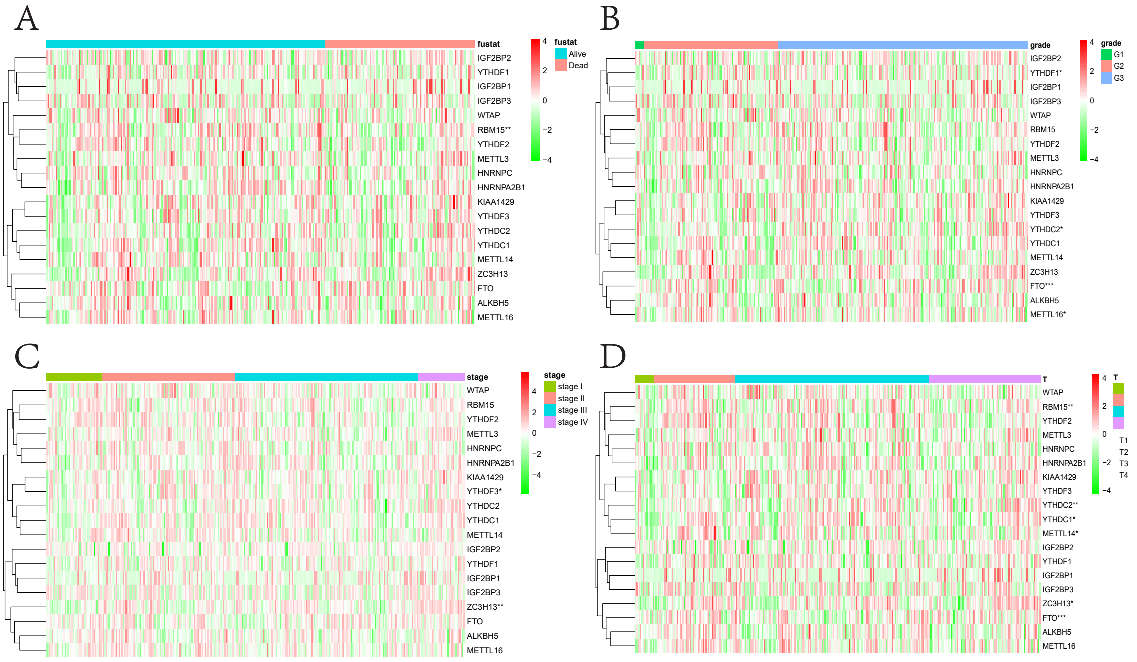

Supplementary Figure 1. Expression of m6A modification genes in GC with different clinicopathological features. (A) fustat status; (B) grade; (C) stage status; (D) T status. *$P < 0.05$, **$P < 0.01$, and ***$P < 0.001$. GC: gastric cancer.

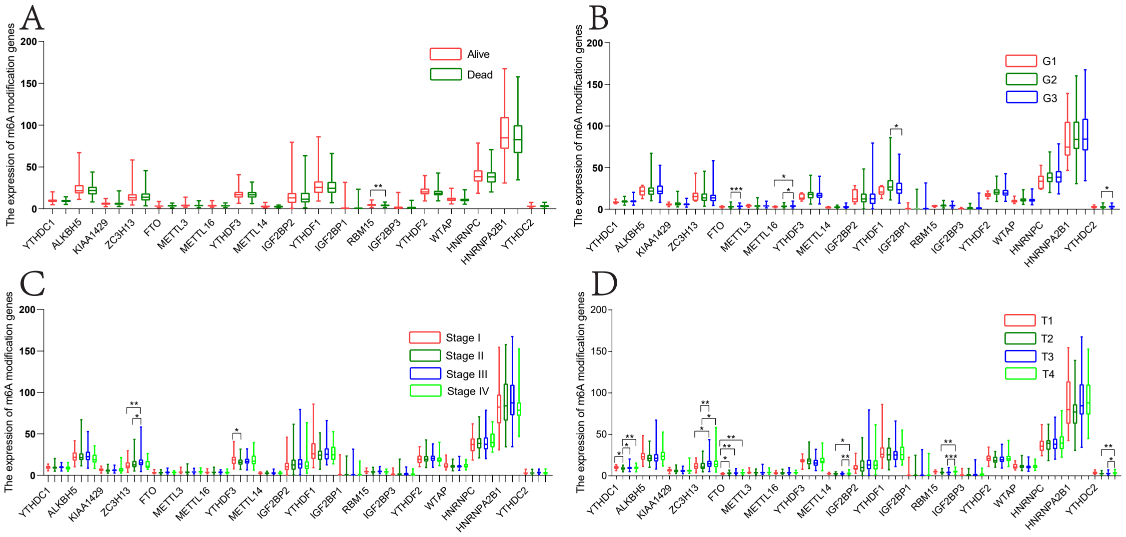

Supplementary Figure 2. Expression of m6A modification genes in GC with different clinicopathological features in the TCGA database. (A) The expression of RBM15 in alive patients was higher than that in dead patients; (B) The expression of FTO, METTL16, YTHDF1, and YTHDC2 were associated with grade; (C) The expression of ZC3H13 and YTHDF3 were related to stage status; (D) The expression of YTHDC1, ZC3H13, FTO, METTL14, RBM15, and YTHDC2 were associated with T status. **P* < 0.05, ***P* < 0.01, and ****P* < 0.001. GC: gastric cancer.

Supplementary Table 1. Basic characteristics of 317 GC patients (from TCGA survival data).

Variables	N (%)/Mean±SD
Age	65±10.62
Gender	
Female	120 (37.9%)
Male	197 (62.1%)
Grade	
G1	7 (2.2%)
G2	108 (34.1%)
G3	202 (63.7%)
Stage	
I	42 (13.2%)
II	101 (31.9%)
III	139 (43.8%)
IV	35 (11.0%)
Topography	
T1	15 (4.7%)
T2	63 (19.9%)
T3	152 (47.9%)
T4	87 (27.4%)
Lymph Node	
N0	99 (31.2%)
N1	83 (26.2%)
N2	69 (21.8%)
N3	66 (20.8%)
Metastasis	
M0	295 (93.1%)
M1	22 (6.9%)

GC: gastric cancer, SD: standard deviation.

Supplementary Table 2. Basic characteristics of 433 GC patients (from GSE84437 survival data).

Variables	N (%)/Mean±SD
Age	60±11.58
Gender	
Female	137 (31.6%)
Male	296 (68.4%)
Topography	
T1	11 (2.5%)
T2	38 (8.8%)
T3	92 (21.2%)
T4	292 (67.4%)
Lymph Node	
N0	80 (18.5%)
N1	188 (43.4%)
N2	132 (30.5%)
N3	33 (7.6%)

GC: gastric cancer, SD: standard deviation.
